# Dysregulated miRNA-375, IL-17, TGF-β, and Microminerals Are Associated with Calpain-10 SNP 19 in Diabetic Patients: Correlation with Diabetic Nephropathy Stages

**DOI:** 10.3390/ijms242417446

**Published:** 2023-12-13

**Authors:** Ghada M. Ezzat, Nashwa Mostafa A. Azoz, Randa A. El Zohne, HebatAllah Abdellatif, Tahia H. Saleem, Wafaa Abdelaziz Emam, Amena Rezk Mohammed, Shimaa Ali Mohamed, Asmaa A. Muhammed, Nessren M. Abd el-Rady, Marwa Hamdy, Hoda S. Sherkawy, Marwa A. Sabet, Salwa Seif Eldin, Marwa A. Dahpy

**Affiliations:** 1 Medical Biochemistry and Molecular Biology Department, Faculty of Medicine, Assiut University, Assiut 71515, Egypt; ghadaezzat@aun.edu.eg (G.M.E.); tahia.h.saleem@aun.edu.eg (T.H.S.); 2 Department of Internal Medicine, Nephrology Unit, Faculty of Medicine, Assiut University, Assiut 71515, Egypt; 3 Department of Clinical Pathology, Faculty of Medicine, Assiut University, Assiut 71515, Egypt; randaz@aun.edu.eg (R.A.E.Z.); hebatallah.abdellatif@aswu.edu.eg (H.A.); 4 Biochemistry Department, Faculty of Medicine (for Girls), Al-Azhar University, Cairo 11351, Egypt; wafaaabdelaziz.medg@azhar.edu.eg (W.A.E.); amenarezk.medg@azhar.edu.eg (A.R.M.); shimaaly.medg@azhar.edu.eg (S.A.M.); 5 Department of Medical Physiology, Faculty of Medicine, Aswan University, Aswan 81511, Egypt; asmaa.ali@med.aswu.edu.eg; 6 Medical Physiology Department, Faculty of Medicine, Assiut University, Assiut 71515, Egypt; 7 Medical Physiology Department, Sphinx University, New Assiut 71515, Egypt; 8 Medical Biochemistry and Molecular Biology Department, Faculty of Medicine, Ain Shams University, Cairo 11591, Egypt; marwahamdy@med.asu.edu.eg; 9 Medical Biochemistry and Molecular Biology Department, Faculty of Medicine, Aswan University, Aswan 81528, Egypt; 10 Department of Microbiology and Immunology, Faculty of Pharmacy, Sphinx University, New Assiut 71684, Egypt; marwasabet@gmail.com; 11 Department of Medical Microbiology and Immunology, College of Medicine, Assiut University, Assiut 71515, Egypt; salwaseifeldein@aun.edu.eg; 12 Department of Basic Medical Sciences, College of Medicine, Princess Nourah Bint Abdulrahman University, Riyadh 11564, Saudi Arabia; 13 Department of Medical Biochemistry and Molecular Biology, Armed Forces College of Medicine (AFCM), Cairo 11774, Egypt

**Keywords:** diabetic nephropathy, miRNA-375, Calpain-10 SNP 19, TGF-β, IL-17, microminerals

## Abstract

Zinc (Zn) and copper (Cu) have been shown to have the potential to improve glucose metabolism through interactions with cytokines and signaling events with multiple genes. miRNA-375 and the Calpin-10 gene are potential genetic biomarkers for the early prediction of diabetic nephropathy (DN). 128 healthy controls and 129 type 2 diabetic (T2DM) participants were matched for age and sex. Three subgroups were identified from the T2DM group: 39 patients had microalbuminuria, 41 had macroalbuminuria, and 49 patients had renal problems. Circulating miR-375 expression levels were measured via qPCR. Calpain-10 SNP 19 (rs3842570) genotyping was assessed with allele-specific PCR in all the included participants. Spectrophotometry was used to measure the concentrations of serum copper, zinc, and magnesium, while ELISA was used to measure the levels of TGF-β and IL-17. There was significant up-regulation in the expression of miR-375 and serum levels of TGF-β, IL-17, Cu, and the Cu/Zn ratio, whereas, in contrast to the control group, the Zn and Mg levels were lower in the T2DM group. The DN groups had significantly lower miR-375, TGF-β, IL-17, Mg, and Zn levels compared with the T2DM without nephropathy group. Furthermore, between TGF-β, IL-17, and miRNA-375, there were notable correlations. Calpain-10 SNP 19 genotype 22 and allele 2 were linked to a higher incidence of T2DM and DN. Significant TGF-β, Cu, Cu/Zn ratio, HbAc1, and creatinine levels, but insignificant miRNA-375 levels, were associated with genotype 22 of Calpain-10 SNP 19. interactions between the Calpain-10 SNP 19 genotype 22 and IL-17, TGF-β, mineral levels, and miRNA-375 might contribute to the aetiology of DN and T2DM and may have clinical implications for diagnosis and management.

## 1. Introduction

Type 2 diabetes mellitus (T2DM) appears to be one of the major diseases threatening to reduce life expectancy for humans around the globe [1,2,3]. Diabetic nephropathy (DN) is a prominent cause of glomerulosclerosis with end-stage renal disease worldwide, with increased mortality among patients with DM [4]. It is responsible for close to half of all chronic kidney disease (CKD) cases. DN usually develops in a genetically susceptible individual because of poor glycaemic control [4]. It is characterised by structural and functional changes, such as the presence of pathological quantities of urine albumin excretion and a lower glomerular filtration rate (GFR) [5].

MicroRNAs (miRNAs) are a class of small, non-coding RNAs of 20–24 nucleotides that play an important role in regulating gene expression. miR-375 is a potential biomarker for the early prediction of T2DM among high-risk individuals. Studies have shown that miR-375 levels are increased in both type 1 and type 2 diabetes [6]. This upregulation is believed to contribute to the pathogenesis of diabetes by affecting pancreatic beta-cell function and insulin secretion [7]. The exact mechanisms by which miR-375 influences diabetes and CKD are still being investigated.

Other genes are the basis for T2DM and DN; one of the genes is calcium-activated neutral protease-10 (CALPIN-10), a gene located at 2q37 that encodes for a non-lysosomal cysteine protease composed of 672 amino acids [8]. The Calpain-10 gene was the first diabetes gene identified by cloning and encoding the Calpain-10 protein [9]. Calpain-10 has an important role in DN as it relates to cellular proliferation and the migration of vascular smooth muscle cells, platelet aggregation, and degranulation [10]. It is involved in the regulation of glucose homeostasis through its actions in pancreatic β cells, liver, skeletal muscle, and adipocytes [11].

The Calpain SNP-19 (rs3842570) SNP is an insertion–deletion polymorphism (HGVS: NC_000002.11: g. 241534262_2 41534293 dup 32) SNP-19 is a two-allele insertion/deletion (indel) polymorphism. Indel-19 (simple tandem repeat insertion/deletion variant with either two or three copies of a 32 bp fragment) is believed to be associated with a high risk of T2DM and an increased BMI [12].

TGF-β is a pleiotropic cytokine and has been recognised as a key mediator of DN [13]. Recently, it has become clear that Th17 immune cells and their effector cytokine, IL-17, represent attractive therapeutic targets in a number of clinical disorders, including renal illnesses. Depending on the cell type and pathological circumstances, IL-17 responses are mostly pro-inflammatory. Numerous miRNAs have been demonstrated to control Th17 cell development in various autoimmune disease models by targeting transcription factors that promote or suppress Th17 differentiation [14].

Zinc is a trace element that is extensively distributed in many organs, and its levels are relatively high in the kidneys [15]. Zinc (Zn) and its transporters are involved in the synthesis and secretion of insulin and the signalling pathways of insulin action [16]. Zn deficiency aggravates diabetes-induced oxidative stress and fibrosis by increasing renal inflammation and reactive oxygen species, thus leading to immune dysfunction. Therefore, zinc deprivation exacerbates kidney damage [17]. Zinc and copper (Cu) compete at the absorption stage. Excess Cu under inflammatory conditions triggers oxidative stress [18].

There are variable interacting etiological factors predisposing for T2DM and/or its complication to DN, so in this study, we aimed, for the first time, to explore the potential relationships of food components and trace elements such as Zn and Cu with inflammatory cytokines biomarkers (TGF-β and IL-17) and the Calpain-10 gene SNP 19 together with the expression of miR-375 in the risk and etiopathogenesis of T2DM without and with CKD complications among Egyptian patients.

## 2. Results

### 2.1. General Characteristics of the Controls and Diabetic Patients

**Figure 1:**
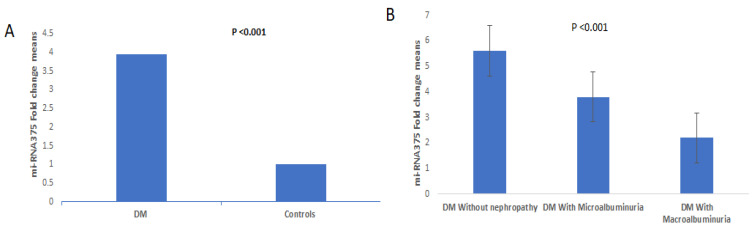
(**A**) Relative quantitative expression of miRNA-375 level in cases and controls. (**B**) Relative quantitative expression of miRNA-375 level in controls and T2DM subgroups.

The clinicopathological data of the diabetic patients and controls who were included in this study are presented in Table 1. The two groups were matched as regards age and gender (*p* > 0.05). The diabetic patients were characterized by a higher BMI than controls and a predominance of hypertension (*p* < 0.001, *p* = 0.003, respectively). In the routine investigations of the T2DM group, the blood glucose, HAc1, and albumin levels were significantly higher in DM patients than in the control group. The relative expression levels of miRNA-375 (Figure 1A) and serum levels of TGF-β and IL-17 showed statistically significant differences between diabetic patients and healthy controls (*p* < 0.001, all). The trace element levels (Zn, Cu, Mg, and Zn/Cu ratio) were statistically significantly increased compared with those in healthy controls (*p* < 0.05 all).

### 2.2. Clinicopathological and Biomarker Levels in Diabetic Subgroups (without Nephropathy, with Microalbuminuria, and with Macroalbuminuria)

The three diabetic subgroups had no statistically significant differences as regards age, sex, and hypertension. The microalbuminuria and macroalbuminuria groups were characterised by statistically significant increases in BMI, glucose, HbA1c, albuminuria, creatinine, urea, eGFR, and Cu/Zn ratio levels when compared with the diabetic group without nephropathy (*p* < 0.05, all). The duration of T2DM was higher in the macroalbuminuria group than in the diabetic without nephropathy and microalbuminuria groups. The diabetic macroalbuminuria group had significantly higher levels of urea, creatinine, and albuminuria than the microalbuminuria group but had statistically significantly lower IL-17 and miRNA-375 levels in comparison with the other two groups and significantly lower TGF-β, Zn, and Mg serum levels when compared with the diabetic group without nephropathy (Table 2 and Figure 1B).

### 2.3. Distribution of the Calpain 10 Gene Polymorphism SNP19 in Controls and Diabetic Patients

**Figure 2:**
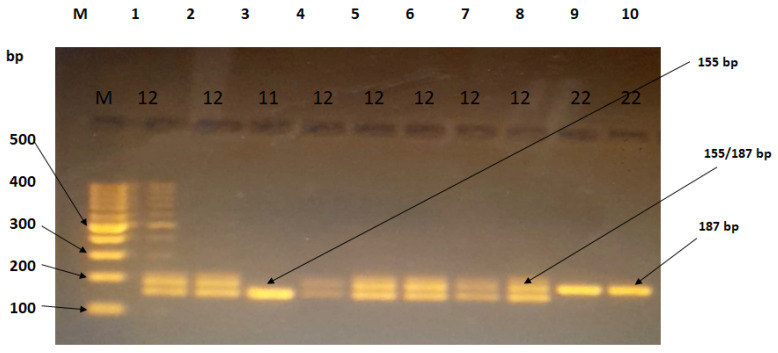
Agarose gel electrophoresis (3%) showing PCR products of SNP-19 (rs3842570). M: DNA ladder of 1000 bp. Lane (3) represents the homozygous genotype (155 bp), lanes (9,10) represent the homozygous genotype (187 bp), and lanes (1,2,4,5,6,7,8) represent the heterozygous genotype (155 bp and 187 bp).

The results of the analysis of the Calpain 10 gene (rs3842570) polymorphism SNP 19 after electrophoresis are presented in Table 3 and Table 4. The genotypes were categorized as wild-type 11 for 155 bp, heterozygous mutant 12 for 187 bp and 155 bp, and pure mutant type 22 for 187 bp, with genotype 11 being the reference genotype Figure 2. This study also examined the dominant and recessive models, as well as individual alleles. The distribution of the genotypes of the investigated SNP followed the Hardy–Weinberg equilibrium.

The present study showed that genotype 22 was associated with an increased risk of diabetes (OR = 5.2, *p* = 0.0009) and diabetic nephropathy (OR = 4, *p* = 0.025) compared with the reference genotype. In the dominant model, which combines the 11 and 12 genotypes, the risk of diabetes was still high (OR = 4.8, *p* = 0.0028) compared with the reference genotype. Similarly, the risk of DN was significantly increased (OR = 3.26, *p* = 0.04). In the recessive model, which combines the 12 and 22 genotypes, the risk of diabetes was significantly increased (OR = 2.4, *p* = 0.0021) compared with the reference genotype, but the risk of DN was non-significantly increased (OR = 1.8, *p* = 0.08) compared with the reference genotype. In terms of individual alleles, allele 1 was significantly associated with a low risk of both diabetes (OR = 1.97, *p* = 0.0004) and DN (OR = 2.4, *p* < 0.0001) compared with allele 2.

### 2.4. Association of Calpain Genotypes with IL-17, TGF-β, miRNA-375, and Routine Laboratory Measurements in Diabetic Patients (n = 129)

The albuminuria, eGFR, TGF-β, IL-17, Zn, Mg, Cu/Zn ratio, and expression of miRNA-375 were not accompanied by significant differences between the three genotypes. Genotypes 12 and 22 had significantly higher HbA1c, urea, and creatinine levels and lower Cu levels than genotype 11, whereas genotype 22 showed higher serum glucose levels than genotype 11 and lower Cu, Cu/Zn ratio, and Zn levels than genotype 12 (Table 5).

### 2.5. Association of Calpain Genotypes with IL-17, TGF-β, miRNA-375, and Routine Laboratory Measurements in Diabetic Nephropathy Patients (n = 80)

There were significant differences in Zn (*p* = 0.027), serum creatinine (*p* = 0.001), Cu/Zn ratio (*p* = 0.033), and HbA1c (*p* = 0.017) levels between the three genotypes. Serum glucose, urea, TGF-β, IL-17, Mg, and miRNA-375 levels were not significantly different between the examined genotypes (*p* > 0.05). Genotype 12 was characterised by a statistically significant increase in glycated Hb, urea, and creatinine serum levels compared with genotype 11. Genotype 22 had higher HBA1c and urea serum levels than genotype 11. A lower Cu level and Cu/Zn ratio were more closely associated with genotype 22 than genotype 12.

The surprising results of our study show an association between genotype 22 and significantly lower urea and creatinine levels in comparison with genotype 12 (*p* < 0.001, both) in the DN group and lower creatinine in diabetic patients (*p* = 0.028) (Table 6).

### 2.6. Correlation between Biomarkers and Routine Diabetic Nephropathy Markers

Table 7 and Table S1 documents the relationships between trace elements and diabetic nephropathy as the serum levels of trace elements, IL-17, and TGF-β were correlated with routine DN laboratory markers. Zn was correlated positively with eGFR and negatively with urea and creatinine levels. In contrast, Cu and Cu/Zn ratio showed significant positive correlations with urea and creatinine and a negative correlation with eGFR and albuminuria. Serum Mg was significantly correlated with all biomarkers, either positively (albumin, eGFR) or negatively (urea, creatinine, albuminuria, glucose, and HbA1c). MiRNA-375, IL-17, and TGF-β showed similar correlational significance as Mg, positively (albumin, eGFR) or negatively (urea, creatinine, albuminuria, glucose, and HbA1c).

## 3. Discussion

Our study aimed to evaluate the role of miRNA-375 in the pathogenesis and progression of diabetes mellitus type 2 (T2DM) and diabetic nephropathy (DN) and to explore its association with the SNP19 gene polymorphism of the Calpin 10 gene. In addition, we aimed to examine their relationships with trace minerals and inflammatory markers in T2DM and DN patients.

MicroRNA-375 (miR-375) has been previously studied for its role in diabetes mellitus (DM) and diabetic nephropathy (DN). However, there are conflicting reports about its levels in T2DM [19]. Our results show that the expression of miR-375 was significantly upregulated in the DM group compared with the control group. Previous studies have suggested that miR-375 plays a critical role in regulating glucose homeostasis, insulin secretion, and pancreatic beta-cell function in DM. One study found that the upregulation of miR-375 in pancreatic beta-cells led to impaired insulin secretion and decreased beta-cell mass, indicating a potential role in the pathogenesis of type 2 diabetes [20]. Another study showed that miR-375 regulates glucose homeostasis by targeting several key genes involved in insulin signalling, including PDK1 and PIK3R1 [21]. Similar to our results, different studies found the upregulation of miRNA-375 in comparison with control subjects [22,23]. Contrary to our results, Raza et al. (2023) [24] documented a lower expression of miRNA-375 in the serum of DM patients. The discrepancy between the previous reports can be explained by Latreille et al.’s (2015) study [25], which showed the upregulation of miRNA-375 after β cells’ destruction by autoantibodies in T1DM. The process by which loss of miR-375 function leads to reduced cell mass is currently unknown and requires further research.

Analysis of the association of miRNA-375 with β-cell functions in our study demonstrated a negative correlation between miRNA-375 and serum glucose and HbAc1 in diabetic and DN patients. This shows that the downregulation of miRNA-375 is related to β-cell function and disorder in glucose metabolism.

García-Jacobo et al. (2019) [26] found opposite results to our study, as they reported that miRNA-375 was not correlated with HbAc1 and was not involved in pancreatic β-cell functionality.

Our study showed that miRNA-375 relative expression levels were decreased in DN groups in comparison with the DM group. miR-375 has been implicated in the progression and pathogenesis of DN and correlated with the progression stage. Studies have shown that dysregulated or decreased miR-375 in the kidneys of diabetic patients may contribute to the development of kidney injury [27]. One study found that miR-375 is an inducing agent of apoptosis through stimulation of P53 or NF-κB in cisplatin-induced kidney damage [28]. Zapała et al. (2023) [29] recorded a lower expression of miRNA-375 in diabetic patients with complications and its control of TGF-β, indicating its involvement in the fibrotic process during DN. Our study demonstrated a positive correlation between miRNA-375 and TGF-β in both DM and DN patients.

The present study identified that the downregulation of miR-375 is involved in the development of DN. This can be explained by findings of a positive correlation of miRNA-375 with levels of eGFR and albumin, which can be confirmed by a direct correlation with Mg and Zn and an indirect correlation with glucose and HbAc1.

IL-17 is involved in the pathogenesis of DN as a pro-inflammatory response in diseases related to obesity and a mediator of autoimmunity and insulin resistance [30]. Both biomarkers were correlated positively with each other and miRNA-375 in the present study. Significantly lower levels were also observed in the DN groups than the DM group, which is similar to the pattern of the reduction in miRNA-375 in the DN group when compared with the DM group [31]. These results emphasise the relationship between miRNA-375 and TGF-β and reveal the role of miRNA-375 and IL-17 in DN. Collectively, we can consider miRNA-375 as a biomarker of inflammation and autoimmunity [32].

The above investigations focused on mir-RNA-375 expression and cytokine action, which may be related to insulin resistance rates that predispose individuals to T2DM and DN later in life. The risk of DM may also be attributed to genetic polymorphisms. The CALPIN-10 polymorphism is one of the most important mutations related to high blood glucose, and its role in the development of diabetic kidney diseases needs meticulous research. Several case–control and association studies have indicated that polymorphisms in CAPN10 are associated with the development of T2DM and insulin resistance [33,34,35].

Our results suggest that genotype 22, as well as allele 2, are associated with an increased risk of diabetes and DN. The recessive model (11 + 12 vs. 22) provided additional evidence for the association between this genotype and the risk of DM, as the recessive model (12 + 22 vs. 11) carries more risk than the dominant model. Bayramci et al.’s study (2017) [33] reported that genotype 22 and allele 2 of CAPN10 SNP-19 were associated with T2DM risk in Turkish patients; however, an earlier study in Turkey found no relationship between Calpain 10 SNP19 and DM but reported an association of genotype 22 with the glycaemic index [34].

The clinical relevance of the results of the Calpain 10 SNP 19 genotype analysis depends on several factors, such as the biomarkers analysed and the patient population studied. In general, the clinical relevance of genetic association studies like this one is determined by assessing whether the observed differences in biomarker levels between the genotypes are significant enough to affect patient outcomes. The present study showed that the significantly higher glucose levels in T2DM patients and higher HbAc1 levels in both T2DM and DN patients were associated with genotypes 22 and 12. A Japanese study reported a similar observation [34].

The significant difference in HbA1c levels between the genotypes in this study could have clinical relevance for patients with diabetes. HbA1c is an important biomarker for monitoring glycaemic control in diabetic patients, and higher levels are associated with an increased risk of complications such as cardiovascular disease and neuropathy. Therefore, if the differences in HbA1c levels between the genotypes observed in this study are clinically significant, they may have implications for the management and treatment of diabetic patients with different Calpain 10 SNP 19 genotypes.

Similarly, the significant difference in creatinine levels between the genotypes could have clinical relevance for patients with kidney disease. Creatinine is a biomarker of kidney function, and higher levels are associated with decreased kidney function. Therefore, the differences in creatinine levels between the genotypes observed in this study are clinically significant and may have implications for the diagnosis and management of kidney disease in patients with different Calpain 10 SNP 19 genotypes.

The significant differences observed in Cu, Cu/Zn ratio, and TGF-β levels in diabetic nephropathy patients with different Calpain 10 SNP 19 genotypes suggest that this genetic variant may play a role in the pathogenesis of DN. Additionally, there are significant correlations between some of the biochemical markers and albuminuria, which is a common complication of DN.

Diabetes mellitus and DN are characterised by disorders influencing mineral levels. Excess Cu and low Zn are linked to redox imbalance and activation of inflammation, which leads to cell death, apoptosis, and diabetic complications [36]. Our results show higher serum Cu and lower serum Zn and Mg levels in diabetic patients, diabetic microalbuminuria, and diabetic macroalbuminuria groups than controls. Consistent with our results, Pouresmaeil et al. found higher serum Cu levels in diabetic patients [37], while Makhlough et al. reported lower serum Cu levels in diabetic patients than controls [38]. Other studies linked higher blood Cu levels in diabetic patients to disorders in the structure of the arterial walls, stress, infection, and dyslipidaemia [39].

A comparison between the diabetic and DN groups revealed significantly higher Cu and lower Zn and Mg levels in DN groups than the diabetic group. The Cu/Zn ratio was insignificantly higher in diabetic patients than controls, whereas the ratio was significantly higher in the macroalbuminuria group than the microalbuminuria group. Therefore, the Cu/Zn ratio may play a crucial role in the development of DKD under inflammatory conditions. The serum Cu/Zn ratio may be a clinically important indicator that enables risk assessment for DKD [1].

The significant difference in the Cu/Zn ratio between the genotypes is also noteworthy. The Cu/Zn ratio is a biomarker that reflects the balance between oxidative stress and antioxidant defence in the body. Higher Cu/Zn ratios are associated with increased oxidative stress, which is a known contributor to the development and progression of diabetic nephropathy. The lack of significant differences in serum TGF-β, serum IL-17, and serum Mg levels between the genotypes suggests that these biomarkers may not be strongly influenced by the Calpain 10 SNP 19 genotype in diabetic nephropathy patients.

Finally, the results of this study provide valuable insights into the potential role of the Calpain 10 SNP 19 genotype in the pathogenesis of diabetic nephropathy. However, further studies are needed to confirm these findings and determine the underlying mechanisms for these associations. Additionally, the clinical relevance of these associations should be further investigated to determine whether they have implications for the diagnosis and management of diabetic nephropathy patients with different Calpain 10 SNP 19 genotypes. We could not find any supporting or conflicting studies as our study is the first study to link Calpain 10 SNP 19 genotyping and trace element metabolism.

## 4. Material and Methods

### 4.1. Ethics Statement

This study was approved by the ethical committee of the Faculty of Medicine, Assiut University (IRP no. 17300181) in accordance with the Helsinki Declaration of 1975, and informed consent was obtained from each patient and control subject before their participation in the study.

### 4.2. Subjects

Three hundred participants were recruited between 2021 and 2022 to the present case–control study; all were Egyptians. Personal and relevant data were collected by means of a well-designed questionnaire. Participants were recruited from the Internal Medicine Nephrology Department, Assiut University Hospital, along with healthy controls. Medical histories were taken, including personal history, family history, history of type 2 diabetes, medication used, presence or absence of hypertension, and presence of other complications associated with diabetes. Exclusion criteria included individuals with cardiac or hepatic affections and those with endocrine disorders, pregnancy, lactation, chronic drug intake, or malignancies. Patients with glomerulonephritis and signs of urinary tract infection were excluded from the study.

According to the inclusion and exclusion criteria, only 257 participants out of the 300 recruited participants were fully enrolled in this study. Their age ranged between 40 and 60 years. They were divided into 129 type 2 diabetic subjects and 128 age- and sex-matched healthy controls. The diabetic group was further subdivided into 3 subgroups according to the associated kidney affection: 49 T2DM patients without kidney complications, 39 T2DM patients with microalbuminuria, and 41 with macroalbuminuria. The diagnosis of type 2 diabetic patients was performed according to WHO criteria, based on fasting plasma glucose (FPG) ≥ 126 mg/dL, and/or 2 h plasma glucose in the 75 g oral glucose tolerance test ≥ 200 mg/dL, random plasma glucose ≥ 200 mg/dL, and HbA1c ≥ 6.5%.

The 24 h urinary albumin and urine albumin/creatinine ratio (ACR) are used to categorise diabetic kidney disease into microalbuminuria and macroalbuminuria. Microalbuminuria is defined as having a 24 h urinary albumin level between 30 and 300 mg/day, while macroalbuminuria as having a 24 h urinary albumin level greater than 300 mg/24 h [40,41]. The patients did not perform intense physical exercise before the albuminuria test.

### 4.3. Sampling and Biochemical Investigations

Six millilitres of antecubital venous blood was obtained from patients and controls after overnight fasting and divided into 3 parts: two millilitres of blood was collected in clean Wassermann empty tubes, left to clot at room temperature, and centrifuged, and serum was separated immediately and stored at −70 °C or was used for measurement of cytokines and minerals with other investigated biochemical parameters.

The leftover 4 mL of blood was collected in ethylene diamine tetra acetic acid (EDTA) anticoagulant tubes and mixed; 2 mL of this was stored at −70 °C for DNA extraction and the other 2 mL was centrifuged at 2500 rpm for 10 min at 4 °C to obtain plasma, which was immediately aliquoted and stored at −80 °C for RNA extraction.

### 4.4. Enzyme-Linked Immunosorbent Assay

An enzyme-linked immunosorbent assay (ELISA) was conducted for TGF-β (Cat no. Sunredbio 201-12-5480), IL-17 (Cat no. Sunredbio 201-12-0143). Micromineral estimation was performed using kits supplied by Bio-diagnostics, El Omraniya, Egypt, for Cu (20 10), Mg (16 10), and Zn (ZN 21 20).

### 4.5. DNA Extraction, Primer In Silico Testing, and Polymerase Chain Reaction (PCR) Allele-Specific Amplification (ASA)

Genomic DNA was extracted from peripheral blood using the Thermo Fisher DNA Mini Kit according to the manufacturer’s instructions (Cat no. K182002).

The primers used for the assessment of Calpain SNP-19 (rs3842570) were designated via in silico testing and were reconstituted using nuclease-free water to a final concentration of 10 μM. The primer sequence was as follows: SNP19: 1 > 2 (NC_000002.12), F: 5-GTTTGGTTCTCTTCAGCGTGGAG-3, and R: 5-CATGAACCCTGGCAGGGTCTAAG-3.

The PCR reaction system included 2.5 mL of PCR buffer solution, 11.3 mL of ddH2O, 2 mL of dNTP, 0.2 mL (5 U/mL) of Taq polymerase, 2 mL of template DNA, 1 mL of each of the upstream and downstream primers.

The PCR conditions were as follows: 5 min of predenaturation at 95 degrees, 30 cycles of denaturation at 95 degrees, annealing at 55 degrees, amplification at 72 degrees, and 10 min of final extension at 72 degrees. The products of SNP 19 were visualised using 2% gel electrophoresis.

### 4.6. RNA Extraction and Quantitative Real-Time Polymerase Chain Reaction (q-PCR)

Following the manufacturer’s recommendations, total RNA was extracted from plasma using QIAzol reagent and the miRNeasy kit from Qiagen (Hilden, Germany). RNA concentration and purity were examined using the Nanodrop technique. The Poly-A-Polymerase enzyme (New England BioLabs, Ipswich, MA, USA) was applied to the poly A tail of the miRNA. cDNA was created using a high-capacity reverse transcription kit (Applied Biosystems, Foster City, CA, USA). Thermo Fischer Scientific’s Maxima SYBR Green qPCR Mastermix was used in a real-time PCR system to assess the expression of mRNA-375 with the negative internal control U6 to normalise expression data. The PCR mixture comprised the following: 12.5 µL of SYBR-Green supermix, 8.5 µL of RNase-free water, 1 µL of each forward and reverse primers, and 2 µL of reverse-transcribed product, for 25 µL in total. Setting a default threshold allowed for the determination of threshold cycle data. The reactive condition consisted of 45 amplification cycles at 95 °C for 5 s, 58 °C for 20 s, and 72 °C for 30 s. The 2^−∆∆Ct^ technique was utilised to determine the relative expression levels of miR-375. The U6 RNA was used as an endogenous reference for comparison with the expression levels of miR-375.

The primers used: miR-375-Forward TTTGTTCGTTCGGCTCGC miR-375-Reverse GCTGTCAACGATACGCTACGT, U6 RNA-Forward CGCTTCGGCAGCACATATAC, and U6 RNA-Reverse TTCACGAATTTGCGTGTCAT [42].

### 4.7. Statistical Analysis

Data analysis was performed using SPSS ver. 20 and GraphPad Prism 5.0 (GraphPad Software, Inc., La Jolla, CA, USA). We used the 2^−∆∆Ct^ technique to analyse the expression levels. For each sample, the threshold cycle (Ct) of fluorescence was first calculated. The expression levels between miR-375 and U6 are shown by the Ct value (∆Ct = Ct miR-375 − Ct U6), and the expression levels between patient samples and the equivalent control are indicated by the ∆∆Ct value (∆∆Ct = ∆Ct patients − ∆Ct control). Finally, the 2^−∆∆Ct^ values (fold change values) were calculated.

## 5. Conclusions

In conclusion, our findings support the belief that miR-375 is involved in the pathogenesis of T2DM and DN. Targeting miR-375 may represent a potential therapeutic strategy for the treatment of these conditions. However, further studies are needed to elucidate the precise mechanisms by which miR-375 contributes to the development of T2DM and DN. In summary, the results suggest that there are significant differences in some biochemical markers between the Calpain 10 SNP 19 genotypes in DN patients. These differences may have clinical implications for the diagnosis and management of diabetic nephropathy patients with different Calpain 10 SNP 19 genotypes. However, it is important to note that genetic association studies like this one are often exploratory in nature and should be followed up with further research to confirm the findings and establish their clinical relevance. Additionally, the clinical relevance of genetic associations may vary depending on the patient population studied and other factors, such as environmental and lifestyle factors.

However, further research studies are needed to confirm the findings and establish their clinical relevance.

## Figures and Tables

**Table 1:** General characteristics, cytokines and minerals serum levels of the controls and type 2 diabetic patients.

	Groups	Controls (No. 128)	T2DM Patients (No. 129)	*p*-Value
Characteristics				
Age (years)		59.1 ± 6.7	58.7 ± 7.8	0.20
Gender n, (%) Males Females		67 (52.3) 61 (47.7)	66 (51.2) 63 (48.8)	0.35
BMI (kg/m^2^)		21.06 ± 3.1	29.5 ± 5.8	**<0.001**
Hypertension n, (%) NO Yes		88 (68.8) 40 (31.3)	64 (49.6) 65 (50.4)	**0.003**
Serum glucose (mg/dL)		80.4 ± 13.3	252.2 ± 66.6	**<0.001**
HbA1c		5.1 ± 0.67	8.1 ± 0.98	**<0.001**
Serum urea (mmol/L)		5.15 ± 1.24	13.6 ± 11.1	**<0.001**
Serum creatinine (μmol/L)		66.6 ± 15.07	222.87 ± 20.5	**<0.001**
Serum albumin (mg/dL)		3.7 ± 0.36	3.2 ± 0.78	**<0.001**
Serum TGF-β (ng/mL)		5.3 ± 0.23	6.4 ± 2.3	**<0.001**
Serum IL-17 (pg/mL)		58.3 ± 10.5	107.9 ± 61.4	**<0.001**
Serum Zn (µg/dL)		74.1 ± 18.4	68.9 ± 15.6	**0.013**
Serum Cu (µg/dL)		186.8 ± 57.3	223.4 ± 42.2	**<0.001**
Serum Mg (µg/dL)		2.2 ± 0.0.7	1.72 ± 0.25	**<0.001**
Cu/Zn ratio		2.7 ± 1.2	3.4 ± 1.1	**<0.001**
miRNA-375 fold change		1 ± 0.24	3.96 ± 2.5	**<0.001**

Data are expressed as means ± SD or numbers, as a percent. The independent sample *t*-test was utilised for comparison between quantitative data, while Chi-square was used for testing the significance between qualitative data. The Mann–Whitney test was used for non-parametric data.

**Table 2:** General characteristics, routine laboratory data, cytokines and minerals serum levels of diabetic patients without nephropathy, with microalbuminuria, and with macroalbuminuria.

	Groups	Diabetic Patients (n = 129)			*p*-Value	P_1_	P_2_	P_3_
Variables		T2DM without Nephropathy (No. 49)	T2DM with Microalbuminuria (No. 39)	T2DM with Macroalbuminuria (No. 41)				
Age (years)		56.56 ± 9.2	58.7 ± 6.5	58.6 ± 6.9	**0.51**	**0.31**	**0.32**	**0.96**
Gender n, (%) Males Females		24 (48.9) 25 (51.1)	16 (41.02) 23 (58.9)	20 (48.8) 21 (51.2)	0.61	0.85	0.63	0.36
BMI (kg/m^2^)		27.04 ± 3.8	31.07 ± 8.2	30.62 ± 2.4	**0.004**	**0.001**	**0.018**	0.35
Hypertension n, (%) No Yes		27 (55.1) 22 (44.9)	21 (53.8) 18 (46.2)	15 (40.6) 26 (59.4)	0.33	0.93	0.33	0.241
Duration of T_2_DM (Years)		6.75 ± 5.3	6.32 ± 4.8	10.46 ± 6.9	**0.003**	0.72	**0.004**	**0.002**
Glucose (mg/dL)		226.2 ± 29.9	259.6 ± 61.8	257.8 ± 75.2	0.061	**0.001**	**0.001**	0.83
HbA1c (%)		6.8 ± 0.41	7.2 ± 0.47	8.3 ± 0.66	**<0.001**	**0.004**	**<0.001**	0.77
Urea (mmol/L)		4.24 ± 1.8	13.58 ± 6.9	23.36 ± 11.9	**<0.001**	**<0.001**	**<0.001**	**<0.001**
Creatinine		70.6 ± 11.1	163.2 ± 77.4	432.8 ± 200.3	**<0.001**	**0.003**	**<0.001**	**<0.001**
(μmol/L)								
eGFR (mL/min/1.73 m^2^)		92.7 ± 7.7	85.3 ± 7.8	76.8 ± 9.04	**<0.001**	**<0.001**	**<0.001**	**<0.001**
Albuminuria		12.2 ± 4.2	96.9 ± 59.7	158.2 ± 15.3	**<0.001**	**<0.001**	**<0.001**	**<0.001**
Serum TGF-β (ng/mL)		7.8 ± 3.2	5.9 ± 1.02	5.3 ± 0.9	**<0.001**	**<0.001**	**<0.001**	0.48
Serum IL-17 (pg/mL)		150.5 ± 81.5	100.8 ± 28.17	68.8 ± 11.9	**<0.001**	**<0.001**	**<0.001**	**0.026**
Serum Zn (µg/dL)		74.5 ± 15.4	65.3 ± 17.7	66.1 ± 11.6	**0.005**	**0.01**	**0.02**	0.93
Serum Cu (µg/dL)		218.5 ± 34.6	201.7 ± 29.2	249.5 ± 46.3	**<0.001**	**0.044**	**<0.001**	**<0.001**
Serum Mg (µg/dL)		1.8 ± 0.21	1.6 ± 0.26	1.66 ± 0.22	**<0.001**	**<0.001**	**<0.001**	0.87
Cu/Zn ratio		3.08 ± 0.9	3.3 ± 1.08	3.9 ± 1.16	**0.001**	0.22	**<0.001**	**0.013**
miR-375 Fold change		5.6 ± 27	3.8 ± 2.2	2.2 ± 1.1	**<0.001**	**<0.001**	**<0.001**	**0.007**

Data are expressed as means ± SD or numbers and percentages. The one-way analysis of variance (ANOVA) test followed by the post hoc test was utilized for the comparison between quantitative data, while Chi-square was used for testing significance between qualitative data. P_1_ indicates significance of diabetic group against microalbuminuria group. P_2_ indicates significance of diabetic group against macroalbuminuria group. P_3_ indicates significance of microalbuminuria group against macroalbuminuria group.

**Table 3:** Genotypes and allele frequencies of Calpain-10 gene SNP-19 (rs3842570) in controls and diabetic patients.

Genotypes	Controls (No. 128)	Diabetic Patients (No. 129)	OR (95%CI)	*p*-Value	Controls (No. 128)	Diabetic Nephropathy (No. 80)	OR (95%CI)	*p*-Value
	n (%)	n (%)			n (%)	n (%)		
11	**71 (55.5)**	**50 (38.8)**	**Reference**	**Reference**	71 (55.5)	27 (33.75)	**Reference**	**Reference**
12	51 (38.9)	57 (44.2)	1.58 (0.9–2.6)	0.08	51 (38.9)	35 (43.75)	1.8 (0.9–3.3)	0.06
22	6 (4.7)	22 (17.1)	5.2 (1.9–13.7)	**0.0009**	6 (4.7)	18 (22.5)	7.8 (2.8–21.9)	**0.0001**
**Dominant** **model**								
11 + 12	122	107	4.8 (1.6–10.7)	**0.0028**	122	62	5.8 (2.1–15.3)	**0.0004**
22	6	22			6	18		
**Recessive** **model**								
11	71	50	2.4 (1.3–4.4)	**0.0021**	71	27	3.2 (1.7–5.9)	**0.0002**
12 + 22	56	79			56	53		
**Allele**								
1	193	157	1.97 (2.8–13.4)	**0.0004**	193	89	2.4 (1.6–3.7)	**<0.0001**
2	63	101			63	71		

**Table 4:** Genotypes and allele frequencies of Calpain-10 gene SNP-19 (rs3842570) in T2DM patients with microalbuminuria or macroalbuminuria and those without nephropathy.

	Without Nephropathy (No. 49)	Diabetic with Microalbuminuria (No. 39)	OR (95%CI)	*p*-Value	Without Nephropathy (No. 49)	Diabetic with Macroalbuminuria (No. 41)	OR (95%CI)	*p*-Value
Genotype	n (%)	n (%)			n (%)	n (%)		
11	24 (47.8)	9 (23.1)	**Reference**	**Reference**	24 (47.8)	18 (45.5)	**Reference**	**Reference**
12	21 (45.7)	15 (38.5)	1.9 (0.69–5.2)	0.2	21 (45.7)	20 (47.7)	1.27 (0.53–3.01)	1
22	4 (6.5)	15 (38.5)	10 (2.6–38.3)	**0.0008**	4 (6.5)	3 (6.8)	1 (0.19–5.03)	
**Dominant**								
11 + 12	45	24	7.03 2.09–23.5	**0.0016**	45	38	0.88 (0.18–4.2)	0.88
22	4	15			4	3		
**Recessive**								
11	24	9	3.2 1.26–8.12	**0.0144**	24	18	1.7 (0.78–3.9)	0.16
12 + 22	25	30			25	33		
**Allele**								
1	69	33	**3.05** **(1.6–5.7)**	**0.0005**	69	56	1.04 (0.54–1.97)	0.90
2	29	45			29	26		

OR: odds ratio; CI: confidence interval.

**Table 5:** Association of Calpain-10 SNP-19 (rs3842570) genotypes with biochemical markers in T2DM patients (n = 129).

	Genotypes	Calpain-10 SNP 19 Genotypes			*p*-Value	P_1_	P_2_	P_3_
Variables		11 (No. 51)	12 (No. 56)	22 (No. 22)				
Glucose (mg/dL)		**246.1 ± 82.6**	**245.7 ± 48.1**	**282.5 ± 59.4**	0.063	0.97	**0.03**	**0.02**
HbA1c (%)		6.8 ± 0.5	7.1 ± 0.6	7.3 ± 0.6	**0.012**	**0.027**	**0.006**	0.25
Urea (mmol/L)		11.18.1 ± 1.4	15.6 ± 1.8	14.2 ± 1.2	0.12	**0.041**	0.29	0.61
Creatinine (μmol/L)		161.5 ± 22.3	289 ± 39.1	172 ± 20.7	**0.005**	**0.002**	0.84	**0.028**
eGFR (mL/min/1.73 m^2^)		86.4 ± 10.5	84.2 ± 11.2	82.01 ± 7.9	0.27	0.31	0.12	0.42
Albuminuria		75.1 ± 9.56	84.1 ± 9.04	87.9 ± 13.8	0.69	0.49	0.46	0.84
Serum TGF-β (ng/mL)		6.7 ± 2.8	6.3 ± 2.4	6.4 ± 2.04	0.73	0.47	0.57	0.97
Serum IL-17 (pg/mL)		121.7 ± 73.5	105.7 ± 59.6	92.5 ± 41.8	0.16	0.19	0.07	0.41
Serum Zn (µg/dL)		68.6 ± 13.8	67.7 ± 15.7	75.3 ± 18.4	0.13	0.71	0.09	0.05
Serum Cu (µg/dL)		222.5 ± 42.6	231.4 ± 43.7	204.7 ± 27.9	**0.042**	0.3	0.09	0.01
Serum Mg (µg/dL)		1.70 ± 0.23	1.75 ± 0.24	1.72 ± 0.26	0.47	0.22	0.53	0.75
Cu/Zn ratio		3.3 ± 0.9	3.6 ± 1.16	2.9 ± 1.16	0.054	0.26	0.12	**0.031**
mRNA-375 fold change		4.1 ± 2.63	4.05 ± 1.8	3.3 ± 2.6	0.42	0.85	0.20	0.24

The data are expressed as means ± SD. The one-way analysis of variance (ANOVA) test followed by the post hoc test was utilised for comparison of means of groups. P_1_: significance of genotype 11 against 12. P_2_: significance of genotype 11 against genotype 22. P_3_: significance between genotypes 12 and 22.

**Table 6:** Association of Calpain 10 SNP 19 genotypes with biochemical markers in T2DM in diabetic nephropathy patients (n = 80).

	Genotypes	11 (No. 27)	12 (No. 35)	22 (No. 18)	*p*-Value	P_1_	P_2_	P_3_
Variables								
Serum glucose (mg/dL)		270 ± 97.5	256.1 ± 83.8	285.8 ± 64.1	0.37	0.46	0.48	0.16
HbA1c (%)		7.06 ± 0.49	7.39 ± 0.49	7.41 ± 0.53	**0.017**	**0.01**	**0.02**	0.90
Urea (mmol/L)		16.9 ± 10.45	22.3 ± **12.3**	15.8 ± 6.8	**0.055**	**0.045**	0.34	**0.001**
Creatinine (μmol/L)		248.07 ± 169.5	431.5 ± **308.9**	189.4 ± **96.6**	**0.001**	**0.003**	0.40	**0.001**
eGFR (mL/min/1.73 m^2^)		79.5 ± 8.3	79.3 ± **10.3**	82.8 ± **7.9**	0.37	0.95	0.18	0.22
Albuminuria		132.3 ± 42.1	127.6 ± 74	104.2 ± 60.4	0.14	0.71	0.06	0.11
Serum TGF-β (ng/mL)		5.4 ± 0.9	5.5 ± 0.9	6.0 ± 4 1.2	0.13	0.59	**0.05**	0.11
Serum IL-17 (pg/mL)		84.5 ± 27.8	85.8 ± 27.5	82.7 ± 24.5	0.92	0.84	0.83	0.16
Serum Zn (µg/dL)		66.8 ± 11.9	**61.4 ± 13.4**	**72.8 ± 19.3**	**0.027**	0.15	0.17	**0.008**
Serum Cu (µg/dL)		231 ± 48.2	233 ± 48.5	205 ± 30.2	0.097	0.95	0.06	**0.044**
Serum Mg (µg/dL)		1.7 ± 0.20	1.6 ± 0.21	1.7 ± 0.24	0.28	0.21	0.78	0.16
Cu/Zn ratio		3.5 ± 1.2	3.9 ± 1.1	3.05 ± 0.08	**0.033**	0.18	0.16	**0.01**
MiRNA-375 fold change		2.8 ± 1.5	3.1 ± 2.2	3.2 ± 1.9	0.74	0.53	0.49	**0.86**

Data are expressed as means ± SD. The one-way analysis of variance (ANOVA) test followed by the post hoc test was utilised for comparison of means of groups. P_1_: significance of genotype 11 against 12. P_2_: significance of genotype 11 against genotype 22. P_3_: significance between genotypes 12 and 22.

**Table 7:** Correlations between biochemical markers in T2DM patients (n = 129).

-		TGF-β	IL-17	Zn	Cu	Mg	Cu/Zn Ratio	miRNA-375 Fold Change
Serum glucose	r	−0.1	−0.1	−0.009	−0.15	−0.708	−0.046	−0.180
	*p*	0.199	0.194	0.920	0.081	**<0.001**	0.606	**0.044**
HBCA1	r	−0.240	−0.262	−0.168	0.068	−0.248	0.134	−0.304
	*p*	**0.006**	**0.003**	0.058	0.443	**0.005**	0.130	**0.000**
eGFR	r	0.189	0.414	0.210	−0.196	0.373	−0.285	0.356
	*p*	**0.033**	**0.000**	**0.017**	**0.026**	**<0.001**	**0.001**	**0.000**
Creatinine	r	−0.273	−0.366	−0.222	0.351	−0.476	0.391	−0.370
	*p*	**0.002**	**0.000**	**0.011**	**0.000**	**<0.001**	**<0.001**	**<0.001**
Serum urea	r	−0.296	−0.402	−0.200	0.220	−0.600	0.293	−0.39
	*p*	**0.001**	**0.000**	**0.023**	**0.012**	**<0.001**	**0.001**	**<0.001**
Serum albumin	r	0.132	0.177	0.146	−0.068	0.403	−0.170	0.285
	*p*	0.137	**0.046**	0.099	0.441	**<0.001**	0.055	**0.008**
Albuminuria	r	−0.416	−0.504	−0.138	0.243	−0.374	0.230	−0.436
	*p*	0.000	0.000	0.119	**0.005**	**<0.001**	**0.009**	**<0.001**
TGF-β	r	1	0.469	0.133	−0.205	0.149	−0.205	0.205
	*p*	-	**0.000**	0.135	**0.021**	0.093	**0.021**	**0.020**
IL-17	r	0.469	1	0.044	−0.151	0.224	−0.151	0.215
	*p*	**0.000**	-	0.623	0.088	**0.011**	0.088	**0.015**
Zn	r	0.133	0.044	1	−0.787	0.185	−0.787	0.180
	*p*	0.135	0.623	-	**0.000**	**0.036**	**0.000**	**0.042**
Cu	r	−0.205	−0.151	−0.787	1	−0.182	1.000	−0.239
	*p*	**0.021**	0.088	**0.000**	**-**	**0.039**	**0.000**	**0.006**
Mg	r	0.149	0.224	0.185	−0.182	1	−0.351	0.258
	*p*	0.093	**0.011**	**0.036**	**0.039**	**-**	**0.000**	**0.003**
Cu/Zn	r	−0.205	−0.151	−0.787	1.000	−0.351	1	−0.239
	*p*	**0.021**	0.088	**0.000**	**0.000**	**0.000**	**-**	**−0.006**
miRNA-375	r	0.205	0.215	0.180	−0.239	0.258	−0.239	1
	*p*	**0.020**	**0.015**	**0.042**	**0.006**	**0.003**	**0.006**	**-**

Correlations were determined with the Pearson correlation test. Two-tailed *p*-values less than 0.05 and less than 0.001 were considered significant. r: correlation coefficient.

## Data Availability

All related data and materials are available on request.

## Supplementary Materials

The supporting information can be downloaded at: https://www.mdpi.com/article/10.3390/ijms242417446/s1.

## Abbreviations

CKD: chronic kidney diseases; Cu: copper; DM: diabetes mellitus; DN: diabetic nephropathy; IL-17: interleukin-17; miRNA-375: microRNA-375; Mg: magnesium; T2DM: type 2 diabetes mellitus; TGF-β: transforming growth factor β; Zn: zinc.
