# Supplementary material for: Dysregulated miRNA-375, IL-17, TGF-β, and Microminerals Are Associated with Calpain-10 SNP 19 in Diabetic Patients: Correlation with Diabetic Nephropathy Stages
Source: Int J Mol Sci. 2023 Dec 13;24(24):17446. doi: 10.3390/ijms242417446 (PMC10744180; doi:10.3390/ijms242417446)
Supplement: Supplementary file 1 [file ijms-24-17446-s001.zip › ijms-2690871-supplementary.pdf]

Table S1. Correlation Heat Map for parameters among patients (n=129).

	Albuminuria	*albumpatients*	*serumalbumin*	*HBA1C*	*glucoseseum*	*seuceatinine*	*egfr*	*BUN*	*snp19genepat*	*tgbfpatients*	*il17patinets*	*znpatients*	*cupat*	*mgpatients*	*cuzratpat*	*micropatien*
Albuminuria	1															
albumpatients	-0.24995	1														
serumalbumin	-0.24995	1	1													
HBA1C	0.476933	-0.19585	-0.19585	1												
glucoseseum	0.273424	-0.17979	-0.17979	0.227791	1											
seuceatinine	0.582014	-0.10625	-0.10625	0.281084	0.064241	1										
egfr	-0.47475	0.160747	0.160747	-0.29286	-0.17833	-0.39828	1									
BUN	0.653598	-0.28323	-0.28323	0.307674	0.159412	0.72545	-0.37477	1								
snp19genepat	0.052067	-0.1702	-0.1702	0.201333	0.205691	-0.06626	-0.12024	0.043959	1							
tgbfpatients	-0.41596	0.132256	0.132256	-0.23958	-0.11441	-0.2731	0.188706	-0.2969	-0.03031	1						
il17patinets	-0.50362	0.176676	0.176676	-0.26193	-0.11569	-0.36641	0.414386	-0.402	-0.1365	0.468854	1					
znpatients	-0.13844	0.146881	0.146881	-0.16801	-0.00987	-0.22293	0.210071	-0.20215	0.170215	0.131628	0.042657	1				
cupat	0.243272	-0.08564	-0.08564	0.068192	-0.09297	0.377717	-0.19555	0.246443	-0.18921	-0.16245	-0.25799	-0.09213	1			
mgpatients	-0.37437	0.168496	0.168496	-0.24818	-0.17365	-0.31269	0.372642	-0.44925	-0.02518	0.14899	0.223634	0.186368	-0.09462	1		
cuzratpat	0.229875	-0.16988	-0.16988	0.133968	-0.04607	0.390687	-0.28501	0.293559	-0.17714	-0.20435	-0.15086	-0.78737	0.632761	-0.1815	1	
micropatien	-0.4439	0.234724	0.234724	-0.29128	-0.17711	-0.36501	0.339469	-0.38789	-0.12037	0.204948	0.214992	0.178827	-0.21083	0.258019	-0.23874	1

	Albuminuria	*albumpatients*	*serumalbumin*	*HBA1C*	*glucoseseum*	*seuceatinine*	*egfr*	*BUN*	*snp19genepat*	*tgbfpatients*	*il17patinets*	*znpatients*	*cupat*	*mgpatients*	*cuzratpat*	*micropatien*
Albuminuria																
albumpatients																
serumalbumin																
HBA1C																
glucoseseum																
seuceatinine																
egfr																
BUN																
snp19genepat																
tgbfpatients																
il17patinets																
znpatients																
cupat																
mgpatients																
cuzratpat																
micropatien																
